# Non-Adherence Rate to Oral Mesalamine in Ulcerative Colitis Patients: A Systematic Review with Meta-Analysis

**DOI:** 10.3390/jpm15040123

**Published:** 2025-03-24

**Authors:** Cristiano Pagnini, Elisabetta Antonelli, Barbara Scrivo, Maria Cappello, Marco Soncini, Roberto Vassallo, Giammarco Mocci, Maria Carla Di Paolo, on behalf of AIGO IBD Commission

**Affiliations:** 1 Azienda Ospedaliera S. Giovanni Addolorata, 00184 Roma, Italy; mcdipaolo@hsangiovanni.roma.it; 2 Ospedale Santa Maria della Misericordia, 06129 Perugia, Italy; elisabetta.antonelli@ospedale.perugia.it; 3 Ospedale “ARNAS Civico—Di Cristina—Benfratelli”, 90127 Palermo, Italy; barbara.scrivo@arnascivico.it; 4 Policlinico “P Giaccone”, 90127 Palermo, Italy; maria.cappello@policlinico.pa.it; 5 Ospedale A. Manzoni ASST, 23900 Lecco, Italy; ma.soncini@asst-lecco.it; 6 Ospedale Buccheri La Ferla, 90123 Palermo, Italy; vassallo.roberto@fbfpa.it; 7 Azienda Ospedaliera “G. Brotzu”, 09134 Cagliari, Italy; giammarco.mocci@aob.it

**Keywords:** inflammatory bowel disease, ulcerative colitis, mesalamine, adherence

## Abstract

**Background/Objectives**: Ulcerative colitis (UC) is a part of inflammatory bowel disease (IBD) and it is characterized by colonic-mucosal chronic inflammation with intermittent clinical activity. Personalized medicine is becoming more and more a relevant method of approach in this field, and the identification of potential concerns in a single patient may contribute to the improvement of the clinical approach. Mesalamine represents the cornerstone of therapy for mild–moderate disease forms, but non-adherence to medical therapy represents a critical health problem, although it is underestimated by many physicians, with evident consequences in terms of disease-related complications. The aim of the present study is to evaluate the magnitude of non-adherence to oral mesalamine in UC patients performing a systematic review and meta-analysis of literature. **Methods**: A literature search in PubMed and Cochrane databases was performed for studies reporting the non-adherence rate to oral mesalamine in adult UC patients, and eligible studies have been selected for evaluation. The type of study (trial vs. observational), geographic area, sample size, method of adherence assessment, and non-adherence rate were considered. **Results**: From a total of 464 articles, 34 studies were included in the meta-analysis after selection. Sixteen studies (47%) are observational, and eighteen (53%) are clinical trials. A total of 12/34 (35%) studies are from North America, 14/34 (41%) from Europe, 4/34 (12%) from Asia, with 4/34 (12%) from mixed areas of the world. The mean non-adherence rate was 32%, but with a consistent variability among the studies. In particular, the non-adherence rate was significantly higher in observational studies vs. clinical trials (47 vs. 20%, *p* < 0.001), and in North American vs. European and Asian studies (54 vs. 23 vs. 4%, respectively, *p* < 0.001). **Conclusions**: The non-adherence rate to oral mesalamine is variably reported in the literature due to the inhomogeneity of available studies, but it represents a consistent problem, often neglected, that deserves future research. A personalized approach by a physician to a single patient can improve the effectiveness of medical therapy and the management of UC patients.

## 1. Introduction

Medication adherence is defined by the World Health Organization (WHO) as “the extent to which a person’s behavior corresponds with agreed recommendations from a health care provider” [1]. Despite significant research efforts and interventions across many therapeutic areas, non-adherence to medical therapy remains a critical health problem, particularly in patients with chronic diseases [2]. Globally, this phenomenon affects around 50% of patients, with evident consequences in terms of disease-related complications, impaired quality of life, and increased healthcare resource utilization [3]. Non-adherence has been linked to higher rates of hospitalizations, disease relapses, and more aggressive therapeutic interventions, factors that negatively impact both patient outcomes and overall healthcare costs [4].

The causes of non-adherence are multifactorial, encompassing patient-related, treatment-related, and healthcare system-related factors [5]. Patient-related factors include forgetfulness, a lack of understanding of the importance of long-term therapy, concerns about side effects, and the erroneous belief that feeling well during remission justifies stopping treatment. On the treatment side, factors such as the complexity of dosing regimens, the frequency of administration, and the cost of medication may hinder adherence. Finally, system-related barriers include limited access to care, inadequate follow-up, and insufficient support from healthcare providers [1].

Ulcerative colitis (UC) is one of the main conditions classified under inflammatory bowel diseases (IBDs), a group of disorders characterized by a dysregulated, immune-driven inflammatory response, primarily affecting the intestine [6]. UC displays an intermittent and relapsing clinical course, with periods of active disease followed by remission, although its exact etiology remains unknown. The hallmark of UC is superficial mucosal inflammation, which starts in the rectum and may extend proximally to the colon [7]. During the active phase, symptoms include diarrhea, bloody stools, urgency, and tenesmus. The primary therapeutic goal is to induce and maintain clinical and endoscopic remission, control the disease, preserve the patient’s quality of life, and prevent complications and ultimately disability [8].

Management strategies for IBD patients are evolving in a double and parallel direction. On the one hand, the standardization of diagnostic and medical procedures, with development and continuous updating of guidelines, aims to guarantee the standard of care for IBD patients in virtually every medical center [9]. On the other hand, the personalized medicine approach, with the holistic consideration of the patient behind the clinical and/or molecular characteristic of the disease, encompassing his/her personal and psychological sphere, is becoming more and more relevant for a truly effective management of such patients [10].

Among the therapeutic options available, mesalamine (5-aminosalicylic acid, 5-ASA) is considered the first-line treatment for both active mild–moderate UC and remission maintenance [11]. Its favorable safety profile and efficacy make it the cornerstone of UC management. Numerous studies and meta-analyses confirm that regular use of mesalamine not only reduces disease symptoms but also delays or prevents the need for therapeutic escalation, such as corticosteroids, immunosuppressants, or biologics [12].

However, as with other chronic therapies, non-adherence to mesalamine poses a major challenge in the long-term management of UC. Non-adherence can significantly compromise the effectiveness of treatment, leading to increased risks of relapse, hospitalization, and complications. In particular, patients who do not adhere to their mesalamine regimen are at a higher risk of disease progression, often requiring more invasive and costly interventions [13]. Previous studies have shown that non-adherence is associated with a substantially higher risk of relapse—up to five times more than in adherent patients [14].

Despite the importance of adherence in managing UC, there are currently no specific guidelines to identify, address, or correct non-adherence in these patients. The lack of clear recommendations is likely due to the variability in findings from studies conducted to date. Some studies report relatively low rates of non-adherence, while others suggest that more than 50% of patients may not adhere to their prescribed therapy [15]. This discrepancy makes it difficult to understand the true scope of the problem and to develop targeted intervention strategies.

Thus, the aim of the present study is to conduct a systematic review of the available literature on non-adherence rates to oral mesalamine in UC patients. The review will be followed by a meta-analysis of the evidence, with the goal of providing a more accurate estimate of the extent of the problem and identifying the main factors associated with the non-adherence rate in the studies. This could provide valuable insights to more accurately identify the magnitude of the problem in order to improve the clinical management of UC patients and optimize the effectiveness of mesalamine therapy, with the final goal to implement the concept of tailoring the therapy for individual patients.

## 2. Materials and Methods

The study was conducted according to the PRISMA statement [16], and the protocol was registered on the PROSPERO website (CRD42024625400). We conducted a literature search for published literature reporting the adherence rate in adult UC patients for oral mesalamine. Three independent researchers conducted a search in the PubMed and Cochrane database with the following strategy: (mesalamine OR mesalamine OR 5ASA) AND (adherence OR compliance). Both clinical trials and observational retrospective studies were considered. The search was not limited to papers in English. Researchers made a final list of eligible papers, progressively excluding citations based on the title, abstract, and full-text reading. Potential disagreements among researchers were resolved by discussion and voting. To be included in the list of examined paper the following criteria had to be fulfilled: the paper has to be published in full text, while abstracts and conference proceedings were excluded; the study should consider only adult patients with UC, while papers also analyzing Crohn’s disease patients or pediatric IBD patients were excluded; the adherence rate should be clearly expressed, and in the case of comparisons between two groups’ adherence (i.e., single vs. multiple mesalamine dose) the mean adherence rate was calculated; studies considering particular sub-groups of patients (i.e., pregnant UC women) were excluded; duplicates or redundant papers were excluded.

For studies comparing adherence in multiple groups (i.e., mesalamine single vs. multiple dose) we calculated the mean of the reported non-adherence rate. For studies reporting the non-adherence rate at different time-points, we considered only the adherence rate of the longest follow-up reported. In case of studies reporting similar cases, we considered only the one with the higher sample size.

For every included study, the type of study (trial vs. observational), geographic area, sample size, method of adherence assessment, and the non-adherence rate were considered. The non-adherence rates were expressed as the percent (%) of considered patients ± standard deviation (SD). We first calculated the total mean of the non-adherence rate of all the studies included in the analysis. Then, a comparison between the different non-adherence rate in studies with the aforementioned characteristics (different kind of study, geographic area, sample size, and method of adherence assessment) was calculated by *t*-test, for the comparison of quantitative parameters between two independent groups. Uni- and multivariate analysis was performed considering the independent variables of high (>50%) and low (<10%) non-adherence rate, and the different study characteristics were considered as binomial dependent variables (trials vs. observational studies, monocentric vs. multicentric, European vs. rest of the world studies, North American vs. rest of the world studies, Asian vs. rest of the world studies, studies with sample size >1000 vs. <1000 subjects, and studies that used questionnaires vs. count of prescription/tablets/refills). Variables that produced a significant result in the univariate analysis were analyzed in the multivariate model. A *p* value of <0.05 was considered significant. Med Calc software (version 12.5) was used for statistical calculation.

## 3. Results

### 3.1. Study Characteristics

**Figure 1:**
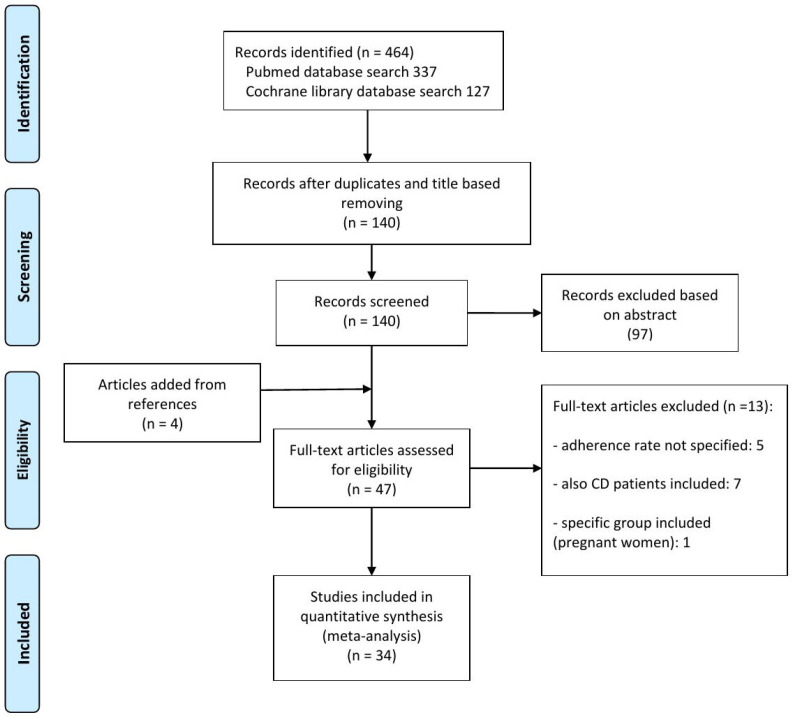
Summary of evidence search and selection for the non-adherence rate for oral mesalamine in adult UC patients.

From an initial result of 464 articles, 324 articles were excluded by only removing duplicates and reading the title, and a further 97 after reading the abstract. Finally, after full text examination, 34 articles were included in the meta-analysis [6,10,11,12,13,14,15,16,17,18,19,20,21,22,23,24,25,26,27,28,29,30,31,32,33,34,35,36,37,38,39,40,41,42] (Figure 1). Regarding study type, 16 studies (47%) are observational, and 18 (53%) are clinical trials. Among the latter, adherence is considered as the primary endpoint in six studies (33%), the non-primary endpoint in three studies (17%), and in nine studies (50%) it is not considered as the endpoint. Considering geographic area, 12/34 (35%) studies are from North America, 14/34 (41%) are from Europe, 4/34 (12%) are from Asia, and 4/34 (12%) are from mixed areas worldwide. Most of the studies are multicentric (24/34, 71%), while consistent variation is observed regarding the method of assessment of adherence: by questionnaire or diary (8/34, 23.5%); by count of prescription/tablets/refills (17/34, 50%); by urinary drug excretion measurements (1/34, 3%); or by multiple methods or not specified (8/34, 23.5%). Characteristics of the included studies are summarized in Table 1.

### 3.2. Non-Adherence Rate

**Figure 2:**
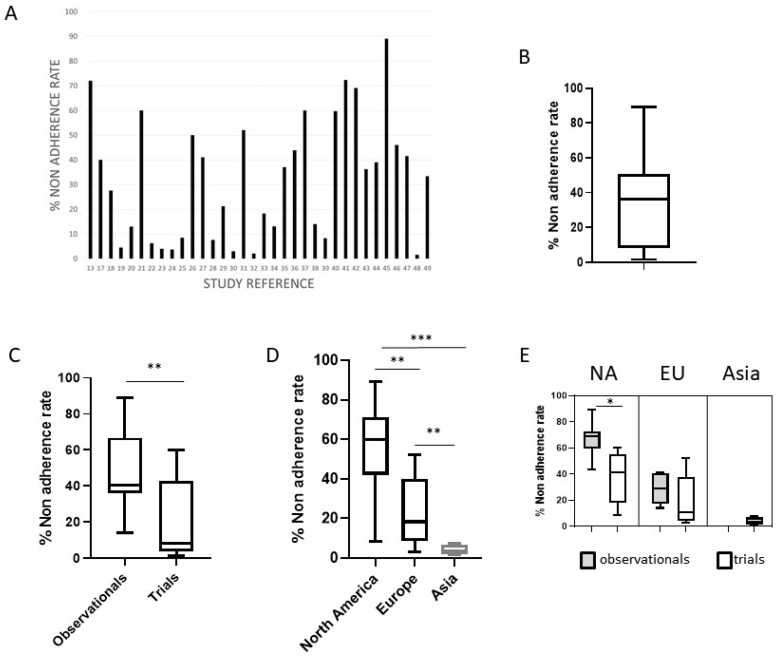
The non-adherence rate in published studies. (**A**) The non-adherence rate (%) in the 34 studies included in the meta-analysis; (**B**) box plot with representation of the upper and lower values, median, and interquartile range for the non-adherence rate in the included studies; (**C**) the significant different non-adherence rate in observational studies vs. trials; (**D**) the significant different non-adherence rate in studies from North America, Europe and Asia; (**E**) considering separately observational studies and trials in the different geographic areas, significant difference emerged in North American studies. NA = North America, EU = Europe, * = *p* < 0.05, ** *p* < 0.001, *** = *p* < 0.0001.

The total mean non-adherence rate was 32.3 ± 24.8%, but consistent variability was observed among the studies (Figure 2A,B). In particular, the non-adherence rate was significantly higher in observational studies vs. clinical trials (46.6 ± 21.6% vs. 19.6 ± 20.5%, *p* < 0.001) and in North American vs. European and Asian studies (54.4 vs. 22.6 vs. 3.9%, respectively, *p* < 0.001) (Figure 2C,D). Considering separately observational studies and trials in the three geographic areas, a significant difference in the non-adherence rate is highlighted in North American studies (67 vs. 34%, *p* < 0.05) (Figure 2E). A non-significant difference has been observed when comparing the non-adherence rate of studies with different methods of adherence evaluation, different study numerosity, and monocentric vs. multicentric studies. At the multivariate analysis, only the geographical area of North America was associated with a high non-adherence rate (>50%) in the studies (*p* < 0.0001), while trial type of the study (*p* < 0.01) and the geographical area of Asia (*p* < 0.005) were both significantly associated with a low non-adherence rate (<10%).

## 4. Discussion

Despite the widespread recognition of non-adherence in chronic disease management, this issue remains particularly overlooked in inflammatory bowel disease (IBD) therapy. A recent web-based survey conducted in the United Kingdom, which included 98 clinicians, revealed that non-adherence in IBD patients was considered an infrequent problem by the majority of respondents (57%). Moreover, only 25% of clinicians regularly screen for adherence, and of those, just 40% use validated tools [50]. These findings reflect the broader clinical misperception regarding the significance of non-adherence in IBD patients. Similarly, an Italian survey conducted by the Italian Association of Hospital Gastroenterologists (AIGO) reported that more than 65% of the 179 hospital-based gastroenterologists surveyed did not consider non-adherence to oral mesalamine in ulcerative colitis (UC) patients to be a significant issue for their own practice (submitted).

This underestimation of the problem is mirrored by the inconsistent data reported in the literature. Studies on non-adherence in UC vary greatly in terms of study design, sample size, methodology, and reported adherence rates, which range from as low as 2% to as high as 72%. Our meta-analysis aimed to address this variability by providing a more precise estimate of the non-adherence rate. We found that the type of study design significantly influenced adherence rates, with clinical trials reporting a much lower mean non-adherence rate compared to observational studies (19.6% vs. 46.8%). This discrepancy is not surprising and not specific for UC patients, as clinical trials typically offer an idealized environment with close patient monitoring and stringent protocols, which may artificially enhance adherence. In contrast, real-world observational studies are subject to numerous confounding factors that can distort adherence measurements, making them more reflective of everyday clinical practice [51]. These real-life studies offer a more accurate portrait of the true burden of non-adherence, providing valuable insights for clinicians who must manage this issue in routine practice.

Another factor that appears to influence non-adherence rate in our meta-analysis is the geographic area of the study. In fact, studies from North America reported significantly higher non-adherence rates compared to European and Asian studies. Indeed, in North America, several variables may contribute to a higher non-adherence rate, such as health insurance related issues and the presence of multiple ethnic groups with potential different adherence trend [52,53]. Moreover, living in a highly industrialized country with more people in a full-time job could induce non-adherent behavior. Indeed, a full-time occupation has been linked to non-adherence in the landmark study by Kane et al. [14]. On the contrary, Asian studies and, in particular, studies from Japan (three-quarters of Asian studies in our meta-analysis), report a high adherence rate, probably reflecting the obedient nature of Japanese people, as previously reported in the literature [49]. Alternatively, the reported difference may be explained by the low numbers of Asian studies, that are all trials, potentially affecting the low non-adherence rate reported. On the other hand, many US studies reported very large observational investigations from health system databases, with a markedly high non-adherence rate reported.

The heterogeneity in adherence rates across studies may also be partly attributed to the diverse methods used to measure adherence [54]. In clinical practice, direct questioning and non-validated questionnaires are often used due to their simplicity. However, these methods are subject to patient reluctance to admit non-adherence and a lack of standardization, which can lead to inaccurate results. More objective methods, such as urine metabolite measurements, though more accurate, are costly and not readily available in most clinical settings. Prescription or tablet count methods, while seemingly straightforward, may not accurately reflect actual drug consumption, as patients can refill prescriptions without consistently taking their medication. The eight-item Morisky Medication Adherence Scale (MMAS-8) is a more formalized tool that has gained traction as a feasible option for adherence assessment [55]. However, its utility in IBD remains debated. For instance, de Castro et al. found that MMAS-8 underperformed when compared to pharmacy refill measurements, with a non-adherence rate of 22.4% versus 37%, respectively, likely due to the tool’s low specificity and negative predictive value [56]. Despite these limitations, MMAS-8 remains one of the most accessible tools for evaluating adherence in clinical practice, offering a pragmatic balance between feasibility and accuracy.

This study aimed to provide a comprehensive evaluation of non-adherence to oral mesalamine in UC patients by synthesizing data from the available literature. For the first time, we attempted to critically analyze the substantial variability in adherence rates reported across studies. While we limited our search to a specific patient population (adult UC patients) and a specific therapy (oral mesalamine), significant heterogeneity persisted, complicating efforts to draw definitive conclusions. When analyzing clinical trials and observational studies separately, some of the variability was reduced, suggesting that study design plays a critical role in determining adherence rates. However, the data remain discrepant, and it is likely that an absolute, universally applicable non-adherence rate is difficult to establish. The observed variability highlights the need for standardized methodologies in future studies, particularly in observational settings where real-world factors may obscure the true extent of non-adherence.

One of the hot topics that has emerged in the last decade in the management of IBD patients is the potential application of personalized medicine; the idea that the therapeutic strategy should ideally be tailored on single patients considering several clinical variables (disease type, location, pattern, comorbidities, etc.) and, in the future, specific molecular biomarkers. Besides the right choice of the drugs available in the market, personalized medicine means the individual approach by the physician to a person, before viewing them as a patient, that comes to our attention for a specific medical problem as they carry with them a personal history and different experiences and attitudes toward the disease that should be necessarily considered and addressed for an effective and positive doctor–patient interaction. The issue of the adherence to medical therapy offers a paradigmatic example of that.

In fact, several factors contribute to non-adherence in UC patients and understanding these is key to developing effective interventions [57]. When a patient is in an active phase of the disease, the discomfort caused by symptoms typically outweighs concerns about the inconveniences of mesalamine therapy, such as the need for constant dosing, potential side effects, or the psychological burden of feeling “sick”. However, once the disease enters remission, these concerns often resurface, leading patients to deprioritize or discontinue their medication. This pattern of behavior is consistent with the five dimensions of non-adherence described by the WHO: social and economic factors, healthcare system-related factors, condition-related factors, patient-related factors, and therapy-related factors. While some of these factors are external and unmodifiable (e.g., socioeconomic status or healthcare access), others—such as patient- and therapy-related factors—can be addressed through targeted interventions. For instance, a strong patient–doctor relationship, characterized by clear communication, empathy, and trust, can significantly improve adherence. Educating patients about the chronic nature of UC and the importance of consistent medication use, even during remission, can foster greater adherence and improve clinical outcomes. Certain sub-groups of patients may be at a higher risk of non-adherence, including younger individuals, those with a recent diagnosis, employed patients, and those in remission [58]. In these populations, regular assessment of adherence and careful exploration of the potential barriers to compliance are crucial for preventing disease flares and maintaining quality of life. Simple strategies, such as patients’ engagement in decision-making, simplifying dosing regimens, and providing tailored educational materials, may mitigate some of the challenges these patients face. Furthermore, adherence should not be assumed but rather routinely assessed, particularly in those at high risk for non-compliance.

The present study confirms that non-adherence to oral mesalamine in UC patients is a more pervasive problem than often recognized. Although the exact magnitude of non-adherence remains difficult to define due to the variability across studies, it is clear that non-adherence rates are likely higher than previously expected. Given the well-documented association between non-adherence and negative clinical outcomes, clinicians must take a proactive approach to addressing this issue. The present work should further underline the importance of personalized medicine in the approach to UC patients; besides guidelines and the standardization of care, the personal and specific physician–patient relationship may still make the difference for optimal management of the disease. Specifically, routine monitoring of adherence should become a standard practice, and clinicians should work collaboratively with patients to identify and overcome barriers to compliance. Simple interventions, such as motivational interviewing, reminder systems, and involving family members in treatment plans, could significantly improve adherence rates.

At the same time, scientific societies and professional organizations have a key role to play in raising awareness about the importance of adherence. These organizations should promote further research into non-adherence, encourage the development of standardized assessment tools, and issue guidelines that support clinicians in managing this issue. Given the complexity of adherence behavior, multifaceted approaches that integrate patient education, healthcare system improvements, and supportive therapies are likely to be the most successful in improving adherence and, consequently, patient outcomes.

## 5. Conclusions

The present study highlights the complexity and significance of non-adherence to oral mesalamine in UC patients. While the true prevalence of non-adherence is difficult to pinpoint, it is evident that this issue deserves greater attention from both clinicians and researchers. Improving adherence should be a priority in the management of UC, as doing so can lead to better disease control, improved patient quality of life, and reduced healthcare costs. In a modern vision of the management of IBD patients, personalized medicine with counseling, and the identification and addressing of specific individual problems can lead to an improvement of the effectiveness of medical therapy, a reduction in disease complications, and a promotion of a higher quality of life.

## Figures and Tables

**Table 1:** Characteristics of the 34 studies included in the meta-analysis.

Study, Year, Ref.	Country	Type	Patient Nr	Assesment Method	Non-Adherence Rate (%)
Mitra, 2012, [13]	USA	Observational multicentric	1693	LifeLink Database	72
Rubin, 2002, [17]	UK	Observational monocentric	363	Prescription count	40
Kane, 2003, [18]	USA	Trial monocentric	22	Validated formula: medication dispensed/days in the refilling interval	27.5
Park, 2019, [19]	Korea	Trial multicentric	180	Self-reported + tablet counts	4.50
Dignass, 2009, [20]	8 European Countries	Trial multicentric	362	Sachet count + validated questionnaire + VAS	12.9
Kane, 2008, [21]	USA	Trial monocentric	20	Pharmacy data	60
Hawthorne, 2012, [22]	UK	Trial multicentric	213	Tablet counts + bottle cap (substudy with 28 + 30 pz)	6.25
Kruis, 2011, [23]	13 European Countries and Israel	Trial multicentric	622	Medication returned + patient diary	4
Kamm, 2008, [24]	19 World Countries	Trial multicentric	362	Pill count	3.7
Prantera, 2009, [25]	Italy, Poland, Ukraine	Trial multicentric	331	Tablet counts	8.5
Ley, 2020, [26]	USA	Trial monocentric	39	Medication possession ratio (MPR)	50
Ballester, 2019, [27]	Spain	Observational monocentric	274	Pharmacy records	41
Yagisawa, 2019, [28]	Japan	Trial monocentric	37	Visual analog scale	7.5
Keil, 2018, [29]	Czech Republic	Observational monocentric	198	Not-validated anonymous questionnaires	21.20
Dignass, 2018, [30]	6 European Countries	Trial multicentric	306	Count of unused medication + patient diary	2.95
Nikolaus, 2017, [31]	Germany	Trial multicentric	248	Morisky Medication Adherence Scale (MMAS)	52
Suzuki, 2017, [32]	Japan	Trial multicentric	604	Diary	2
Algaba, 2017, [33]	Spain	Observational multicentric	203	Morisky–Green scale	18.2
Kane, 2012, [34]	UK	Trial multicentric	208	TrialCard prescription refill data	13
Moshkovska, 2009, [35]	UK multicentric	Observational multicentric	169	Self-report data and urinary drug excretion measurements (*n* = 151)	37
Kane, 2008, [36]	USA	Observational multicentric	4313	Pharmacy records	43.8
Kane, 2001, [37]	USA	Observational monocentric	94	Refill information from computerized pharmacy records	60
Pedersen, 2014, [38]	Denmark	Observational multicentric	95	Visual Analog Scale (VAS) and the Medical Adherence Rating Scale (MARS)	14
Gillespie, 2014, [39]	USA	trail substudy monocentric	58	Self-report, tablet counts, medication event monitoring system	8.3
Khan, 2012, [40]	USA	Observational multicentric	13062	Medication possession ratio (MPR), continuous single-interval medication availability (CSA) and continuous multiple-interval medication gaps (CMG).	59.7
Lachaine, 2013, [41]	Canada	Observational multicentric	1681	RAMQ pharmaceutical services database	72.3
Yen, 2012, [42]	USA	Observational multicentric	5664	IMS LifeLink Health Plan Claims Database	69
Schreiber, 2013, [43]	Canada and Europe	Observational multicentric	775	Patient-reported adherence survey	36.2
Hodgkins, 2012, [44]	USA, UK, Germany, and Canada	Observational multicentric	400	MMS	39
Kane, 2011, [45]	USA	Observational multicentric	44,191	Pharmacy database prescription and refill records	89
Moshkovska, 2011, [46]	UK	Trial monocentric	71	Determination of 5-ASA and N-acetyl-5-ASA concentration in the urine samples	46
Moss, 2010, [47]	USA	Trial multicentric	81	Refill data from pharmacies	41.5
Watanabe, 2013, [48]	Japan Multicentric	Trial multicentric	301	Not specified	1.5
Kawakami 2022, [49]	UK and Japan	Observational multicentric	532	MMAS-8	33.3

## Data Availability

Data available upon request.

## Abbreviations

The following abbreviations are used in this manuscript:

IBD	Inflammatory bowel diseases
UC	Ulcerative colitis
5-ASA	5-aminosalicylic acid
MMAS	Morisky Medication Adherence Scale
